# 

**Published:** 1884-04

**Authors:** 


					﻿SURG  GEWhidq
Vol. XX.
April, 1884.
No. l.\
ThE
BISTOURY
Thad S Up de Graff M.D
EDITOR
ELMIRA.N.Y.
				

